# Advances in adoptive cell therapy for ovarian cancer

**DOI:** 10.3389/fimmu.2026.1794716

**Published:** 2026-04-27

**Authors:** Na Zhao, Yujiao An, Shanwei Guo, Zhen Xu, Hongtang Shi, Zhentao Zhang

**Affiliations:** 1 Department of Obstetrics and Gynecology, Affiliated Hospital of Binzhou Medical University, Binzhou, China; 2 Operating Room, Department of Anesthesiology, Affiliated Hospital of Binzhou Medical University, Binzhou, China

**Keywords:** adoptive cell therapy, chimeric antigen receptor T cells, immunosuppression, ovarian cancer, tumor immune microenvironment, tumor-infiltrating lymphocytes

## Abstract

Ovarian cancer remains the most lethal gynecological malignancy worldwide, with late-stage diagnosis, high recurrence rates, and chemoresistance posing persistent clinical challenges. Adoptive cell therapy (ACT), a rapidly advancing immunotherapeutic strategy, offers promising efficacy with low systemic toxicity and has emerged as a compelling option to address these limitations. This review provides a comprehensive overview of ACT modalities—including tumor-infiltrating lymphocytes (TILs), chimeric antigen receptor T cells (CAR-T), natural killer (NK) cells, and other emerging cellular therapies such as TCR-T, cytokine-induced killer (CIK) cells, and γδ T cells—in the context of ovarian cancer. We highlight the mechanistic underpinnings of ACT, the immunosuppressive features of the ovarian tumor microenvironment, and cutting-edge advances in combinatorial regimens, genetic engineering, and cell design aimed at overcoming therapeutic resistance. In particular, we discuss antigen specificity, tumor immune evasion, and stromal barriers, and summarize current clinical trial progress, efficacy outcomes, and translational barriers. Together, these insights underscore the transformative potential of ACT in ovarian cancer and outline future directions for personalized and scalable immunotherapies.

## 1 Introduction

Ovarian cancer is one of the three major malignancies of the female reproductive system and remains a leading cause of gynecological cancer-related mortality worldwide (1). At present, the standard therapeutic approach for ovarian cancer is centered on cytoreductive surgery followed by platinum-based adjuvant chemotherapy (2). Although this regimen can achieve favorable initial responses in a proportion of patients, its overall clinical benefit remains constrained by several persistent challenges (3). Most patients are diagnosed at an advanced stage, at which extensive intraperitoneal dissemination and a high tumor burden frequently preclude complete surgical resection (4). Moreover, despite standard treatment with cytoreductive surgery and chemotherapy, the majority of patients experience relapse within 2 years, often accompanied by the development of chemoresistance, which ultimately drives disease progression and contributes to poor survival outcomes (5).

In addition, a subset of patients are not suitable candidates for surgery because of widespread metastasis or compromised physical status (6, 7). These limitations underscore the urgent need to develop novel therapeutic strategies that are more effective, less toxic, and capable of improving the long-term prognosis of patients with ovarian cancer (8). Immunotherapy has emerged as the fourth major pillar of cancer treatment, alongside surgery, chemotherapy, and radiotherapy (9–12). Broadly, immunotherapy includes immune checkpoint inhibitors (ICIs), adoptive cell immunotherapy (ACT), cytokine therapy, and cancer vaccines (13). Among these, ACT is considered one of the most promising immunotherapies due to its high efficacy and low toxicity (14). This review summarizes the mechanisms of ACT and the latest advances in its application to ovarian cancer, offering insights for optimizing clinical treatment strategies and guiding future research directions for innovative therapies.

## 2 Adoptive cell therapy

### 2.1 Overview of adoptive cell therapy

Adoptive cell therapy represents a paradigm-shifting immunotherapeutic modality wherein autologous or allogeneic immune cells are harvested from patients, subjected to ex vivo expansion and functional reprogramming, and subsequently reinfused to potentiate specific tumor recognition and cytolysis (15). By increasing the immunogenicity of tumor cells and reversing the immunosuppressive microenvironment, ACT stimulates and sustains the body’s long-lasting anti-tumor immune response (16). Clinically, ACT has demonstrated profound efficacy across diverse malignancies. For instance, chimeric antigen receptor T-cell therapy (CAR-T) has achieved a complete response rate (CRR) of over 80% in patients with acute lymphocytic leukemia (ALL) (17). However, the therapeutic performance of ACT differs substantially between hematologic malignancies and solid tumors. In hematologic cancers, target antigens are often more uniformly expressed, malignant cells are more readily accessible in the circulation or bone marrow, and infused effector cells encounter fewer physical barriers to trafficking and target engagement (18, 19). Solid tumors are characterized by substantial intertumoral and intratumoral antigen heterogeneity, spatially restricted immune infiltration, and a highly suppressive tumor microenvironment that collectively constrain the persistence, penetration, and cytotoxic efficacy of adoptively transferred cells (20, 21). These constraints are particularly relevant in ovarian cancer, where stromal desmoplasia, aberrant vasculature, immunosuppressive cytokines, and inhibitory immune cell populations create a hostile niche for ACT (22, 23). Thus, although ACT has transformed the treatment landscape of hematologic malignancies, its successful translation to ovarian cancer requires additional strategies to address antigen escape, poor tumor homing, and immune suppression (22, 24). Emerging non-invasive therapeutic approaches may provide an important means of augmenting ACT responsiveness. The thermal effect induced by non-invasive strategies can enhance T-cell infiltration into tumors and the killing effect on tumor cells in melanoma and orthotopic liver cancer mouse models during ACT treatment (25). Similarly, cryo-thermal therapy can remodel the tumor immune microenvironment, significantly improving the efficacy of ACT (26). These treatment methods not only enhance the specific recognition and killing ability of immune cells against tumors but also overcome immunosuppression in the tumor microenvironment.

### 2.2 Functional characteristics of NK cells

ACT can be divided into HLA-dependent ACT and HLA-independent ACT. HLA-dependent ACT recognizes tumor antigens presented by HLA molecules through T-cell receptors, including T-cell receptor engineered T-cell therapy (TCR-T) and tumor-infiltrating lymphocyte therapy (TIL) (27). The latter recognizes and kills tumor cells through HLA-independent mechanisms, including CAR-T, NK cell therapy, and other HLA-independent ACT therapies (28). The antitumor activity of ACT is mediated by the *in vivo* persistence and function of ex vivo expanded or genetically engineered immune cells, with therapeutic efficacy largely determined by their capacity for activation, tumor-specific recognition, proliferation, and cytotoxic effector responses (29–31). T cells are genetically engineered to acquire the ability to efficiently recognize tumor antigens via expressing chimeric antigen receptors, CAR, or specific T cell receptors. CAR-T cells directly recognize tumor cell surface antigens (such as CD19 and MUC16) through single-chain variable fragments (scFv), while TCR-T cells recognize intracellular antigen peptides presented by MHC molecules (32, 33). This recognition event triggers intracellular activation programs that drive cytotoxic responses, including the release of perforin and granzymes, engagement of death-receptor pathways, and the production of effector cytokines such as IFN-γ and TNF-α, thereby promoting both direct tumor-cell killing and broader immune recruitment. In contrast to T-cell-based ACT, NK cells exert antitumor activity in a non-antigen-specific, MHC-independent manner through “missing-self” surveillance together with signals derived from cellular stress ligands (29).

**Figure 1:**
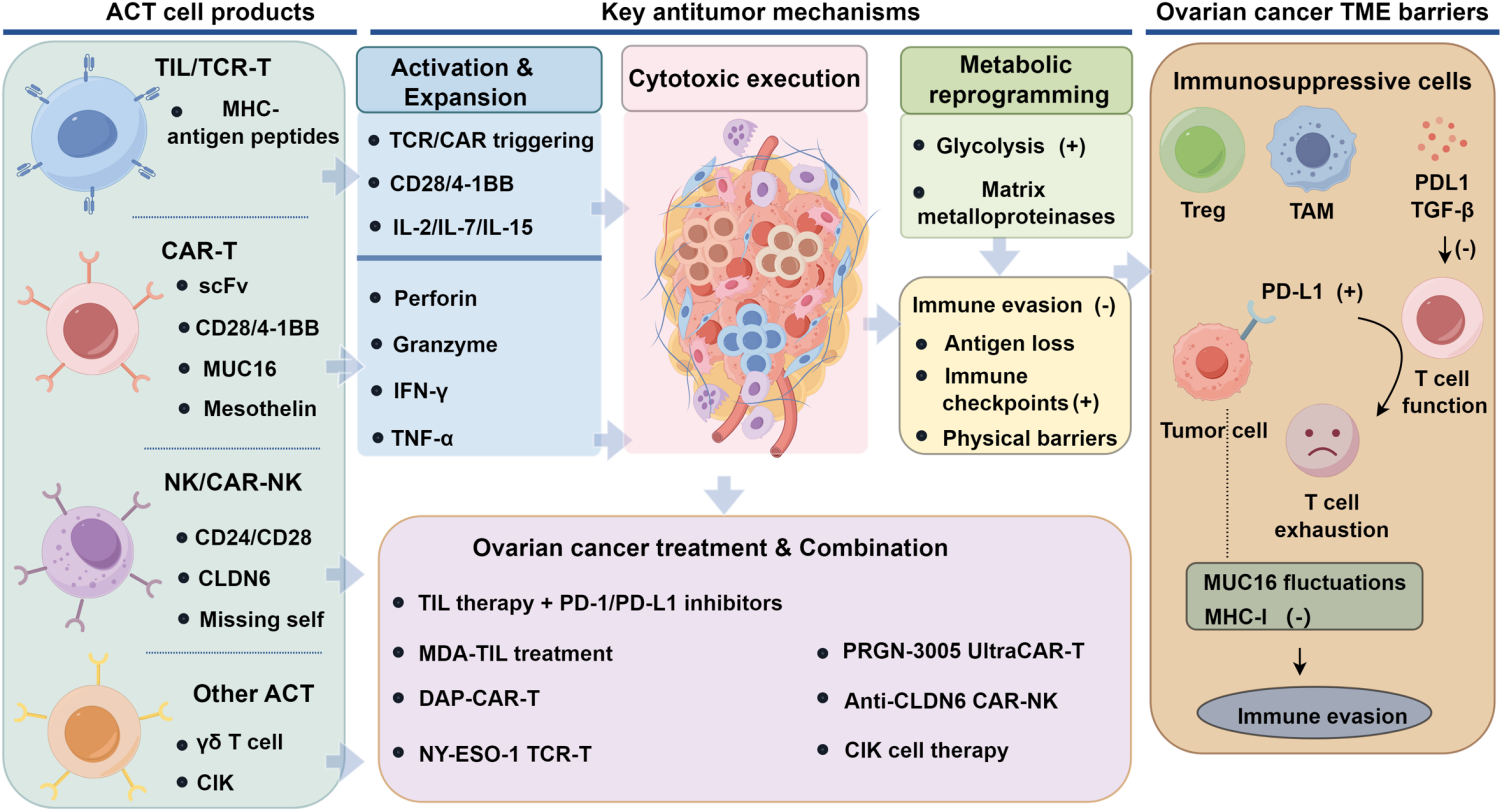
Mechanisms of tumor cell recognition and targeting action of ACT.

However, the efficacy of ACT is frequently constrained by the immunosuppressive tumor microenvironment. Immunoregulatory cell populations, including regulatory T cells and tumor-associated macrophages, together with inhibitory mediators such as PD-L1 and TGF-β, can markedly impair effector cell activation, infiltration, persistence, and cytotoxicity (16, 34). To overcome these barriers, ACT enhances T cell proliferation and anti-exhaustion capabilities by introducing costimulatory molecules (CD28 and 4-1BB), while combining immune checkpoint inhibitors (PD-1/PD-L1 blockers) to reverse the immunosuppressive state (35). Additionally, ACT can improve T cell adaptability and infiltration in the tumor microenvironment by targeting metabolic reprogramming to enhance glycolytic capacity and degrading the tumor stromal barrier through secretion of matrix-remodeling approaches, thereby effectively countering tumor immune evasion mechanisms such as antigen loss, upregulation of immune checkpoints, and physical barriers (36). Overall, ACT enhances T cell anti-tumor activity through multi-layered mechanisms, remodels the tumor microenvironment, and provides new strategies to overcome tumor immune evasion, demonstrating significant clinical potential (**Figure 1**).

### 2.3 Ovarian cancer immune microenvironment for adoptive cell therapy

The immunosuppressive microenvironment of ovarian cancer has unique pathological features that constitutes a major barrier to the efficacy of ACT. Inhibitory immune cells such as regulatory T cells and tumor-associated macrophages account for 30% of the immunosuppressive microenvironment, directly suppressing T cell function through IL-10 and TGF-β (37). Immune checkpoints like PD-L1 are overexpressed in 60% of advanced ovarian cancer patients, accelerating T cell exhaustion (15, 38). Tumor antigen heterogeneity, including dynamic fluctuations in MUC16 expression, undermines stable target recognition, while downregulation of major histocompatibility complex class I (MHC-I) further compromises immune surveillance and facilitates escape from effector-cell-mediated killing (39). Besides, dense fibrotic stroma and abnormal vascular networks hinder cell infiltration (40). To address the major translational barriers facing ACT in ovarian cancer, current efforts are centered on refining tumor-targeting specificity and reinforcing T-cell functional competence (22, 41). Representative strategies include dual-antigen CAR-T systems targeting molecules such as MUC16 and mesothelin, together with neoantigen-informed TCR-T designs intended to broaden tumor recognition while minimizing immune escape (33). Concurrently, next-generation engineering approaches seek to improve T-cell persistence and effector durability through optimized co-stimulatory signaling, particularly 4-1BB-based constructs, and metabolic reprogramming strategies that support mitochondrial integrity and long-term cellular fitness within the hostile tumor microenvironment (42). Immunosuppression reversal—combining PD-1 inhibitors or A2aR antagonists (CPI-444) to block inhibitory signals (43).

## 3 Research progress of adoptive cell therapy in ovarian cancer

### 3.1 TIL in ovarian cancer

TIL therapy is an immunotherapeutic approach that involves isolating lymphocytes from tumor tissue, expanding them in a culture medium enriched with IL-2 to obtain a large number of highly purified TILs, and then reinfusing them into the patient to enhance the antitumor activity of immune cells (44, 45). In ovarian cancer, interest in TIL-based therapy has increased steadily. Early clinical trials have confirmed the preliminary efficacy of TIL therapy. Aoki et al. (46) reported an objective response rate (ORR) of 71% with monotherapy in advanced patients, while combination with chemotherapy further increased the ORR to 70%. In postoperative adjuvant therapy, TIL therapy achieved higher overall survival than the control group (47). In recent years, research on TIL therapy has increasingly focused on overcoming the immunosuppressive tumor microenvironment and exploring combination strategies. TIL combined with IL-2 treatment could stabilize or shrink target lesions in platinum-resistant patients, though the emergence of new lesions indicated incomplete reversal of local immunosuppression (48). To enhance efficacy, the combination of TIL therapy with immune checkpoint inhibitors (PD-1/PD-L1 inhibitors) is being widely investigated. A clinical trial in breast cancer patients demonstrated that TIL combined with PD-1 inhibitors significantly improved ORR with manageable safety. Additionally, combinations of TIL therapy with chemotherapy or targeted therapy are under exploration, showing promising synergistic effects (49). Currently, multiple clinical trials are further validating the efficacy and safety of TIL therapy. For example, in the NCT03610490 trial, autologous MDA-TIL treatment in platinum-resistant patients resulted in a median overall survival of 4.9 months and a disease control rate (DCR) of 67% (50).

### 3.2 CAR-T in ovarian cancer

A key aspect of CAR-T therapy is identifying ideal tumor-specific antigens. Currently, clinical studies targeting various CAR recognition sites in cancers are underway, including FRα (NCT00019136) (51), EGFR (NCT01869166) (52), and HER2 (NCT01935843) (53). Additionally, new targets such as CD44, CD133, and ALDH—markers associated with ovarian cancer stem cells—as well as surface markers of cancer-associated fibroblasts (CAF) and tumor vascular endothelial cells in the tumor microenvironment, are gradually gaining attention (54). Exploration of these targets will provide new directions for optimizing CAR-T therapy in ovarian cancer treatment. In terms of clinical research, advanced ovarian cancer patients with platinum-resistant showed that 67% of patients experienced reduced tumor burden, with a disease control rate of 86% (55). It is reported that DAP-CAR-T cells targeting mesothelin achieved a 100% disease control rate in six patients with mesothelin (MSLN)-positive advanced recurrent refractory ovarian cancer, including two cases of partial response and four cases of stable disease, with a median overall survival of 11 months (56). To further improve the efficacy of CAR-T cells in ovarian cancer, researchers have optimized the structure and function of CAR-T cells in multiple ways. CAR-T cell therapy targeting tumor-associated glycoprotein-72 (TAG-72) demonstrated significant anti-tumor efficacy in a mouse model, with a complete response rate of 40% (57). The addition of IL-12 helped T cells kill tumor cells, enter the bloodstream, and eliminate distant metastases. Meanwhile, the rational use of cytokines can significantly enhance CAR-T cell function. For example, CAR-T cells with a memory phenotype generated through short-term co-culture with IL-2 exhibited greater persistence and anti-tumor activity *in vivo* (58). IL-15 and IL-7 promote the *in vitro* expansion of CAR-T cells and enhance their anti-tumor capability. Inhibiting IL-10 in the tumor microenvironment can reverse immunosuppression, improving the survival and anti-tumor effects of CAR-T cells (59). Researchers have developed bispecific CAR-T cells that can simultaneously recognize two different tumor antigens, such as MUC16 and mesothelin, thereby reducing the likelihood of tumor escape (33).

Additionally, combination therapy is an important strategy to improve the efficacy of CAR-T. Studies indicate that combining CAR-T cells with immune checkpoint inhibitors (PD-1/PD-L1 inhibitors) can reverse the immunosuppressive state of the tumor microenvironment and enhance T cell activity (60, 61). CAR-T cells combined with the anti-angiogenic drug apatinib reduced tumor lesions and lowered CA125 levels, suggesting the potential value of combination therapy in ovarian cancer (62). Furthermore, while radiotherapy and chemotherapy inhibit T cell function, they can also expose more tumor targets, thereby enhancing the therapeutic effect of CAR-T cells. For example, the combination of paclitaxel and HER2-CAR-T therapy exhibits synergistic anti-tumor effects. This strategy not only directly kills tumor cells but also creates a more favorable treatment environment for CAR-T cells by altering the tumor microenvironment, offering more possibilities for ovarian cancer treatment (63, 64). Despite substantial progress in CAR-T therapy for ovarian cancer, several major challenges continue to limit its broader clinical translation. First, the marked heterogeneity of ovarian cancer and its profoundly immunosuppressive tumor microenvironment can impair T-cell trafficking, persistence, and cytotoxic function, thereby reducing therapeutic efficacy (65, 66). Second, treatment-related toxicities remain an important clinical concern. Among these, cytokine release syndrome (CRS) is one of the most recognized immune-mediated adverse events following adoptive cell therapy (15, 67). CRS is primarily driven by rapid immune activation after CAR-T-cell engagement with tumor cells, resulting in excessive production of inflammatory mediators such as IL-6, IL-1, IFN-γ, TNF-α, and granulocyte-macrophage colony-stimulating factor (GM-CSF) (68). This inflammatory cascade may induce fever, hypotension, capillary leakage, coagulopathy, and, in severe cases, multiorgan dysfunction. In solid tumors such as ovarian cancer, CRS appears to be generally less frequent and often less severe than that observed in hematologic malignancies, likely because of lower circulating tumor burden, restricted CAR-T expansion, and physical barriers within the tumor microenvironment (69, 70). Nevertheless, CRS remains clinically relevant, particularly as newer CAR designs, regional delivery strategies, and combination regimens enhance T-cell activation and intratumoral accumulation (71, 72). More importantly, identifying highly expressed specific targets in tumors is key to further enhancing the effectiveness of CAR-T therapy.

### 3.3 NK cell therapy in ovarian cancer

NK cell therapy has emerged as a promising ACT strategy in ovarian cancer. In preclinical models, IL-15-based approaches enhance NK-cell activation, increase granzyme and IFN-γ release, and improve control of intraperitoneal ovarian tumors (73). In a recent phase I trial of intraperitoneally infused stem-cell-derived allogeneic NK cells in recurrent epithelial ovarian cancer, treatment was well tolerated without severe toxicity, and 5 of 7 patients showed transient reductions in CA125 levels, with one patient achieving radiologically stable disease and a progression-free survival of 9 months (74). Earlier translational clinical data summarized in recent reviews also suggest that responses to allogeneic NK-cell infusion in ovarian cancer are heterogeneous, ranging from temporary disease stabilization to progressive disease, underscoring the need for further optimization of cell source, dosing schedule, and microenvironmental support (75). Additionally, another clinical trial assessed the activity of intraperitoneally infused expanded allogeneic NK cells in ovarian and fallopian tube cancers, with promising results anticipated. With advancements in genetic engineering, chimeric antigen receptor NK (CAR-NK) cells have become a research hotspot. designed a dual CAR-NK cell targeting CD24 and CD28, which not only directly eliminates ovarian cancer cells but also exhibits strong cytotoxicity against ovarian cancer stem cells (76). In parallel, engineered NK-cell platforms are gaining momentum. Preclinical studies have shown that anti-CLDN6 CAR-NK cells directly killed ovarian cancer cells *in vitro* and effectively reduced tumor growth and metastasis in subcutaneous and intraperitoneal ovarian cancer animal models (77). Currently, human embryonic stem cells and induced pluripotent stem cells are the primary sources of NK cells in clinical trials, and their scalable cultivation techniques provide a foundation for clinical translation.

### 3.4 Other types of adoptive cell therapy in ovarian cancer

In addition to TIL, CAR-T, and NK cell therapies, TCR-T cell therapy involves genetically engineering T cell receptors to recognize tumor antigens presented by MHC molecules. Compared to CAR-T, TCR-T can target intracellular antigens (NY-ESO-1, MAGE-A), thereby expanding the range of target selection (78, 79). The results of a Phase I/II clinical trial on TCR-T cell therapy showed that the ORR reached 55% in ovarian cancer patients treated with NY-ESO-1 TCR-T, with some patients achieving long-term disease stabilization (80). Additionally, cytokine-induced killer (CIK) cell therapy involves the *in vitro* culture of peripheral blood mononuclear cells induced by cytokines to produce a heterogeneous cell population with characteristics of both NK cells and T cells (81). The clinical application of CIK cells in ovarian cancer primarily focuses on combination with chemotherapy or targeted therapy. Studies have shown that CIK cell therapy has a high safety profile but relatively weak efficacy and lacks specificity (82). γδ T cell therapy utilizes a special subset of T cells that recognize tumor cells in a non-MHC restricted manner (83, 84). The potential application of γδ T cells in ovarian cancer has garnered significant attention, particularly for their effect on cancer stem cells. However, the expansion of γδ T cells is challenging, and their efficacy requires further validation (85) (**Table 1**).

**Table 1:** Comparison of major adoptive cell therapy modalities in ovarian cancer.

ACT modality	HLA dependence	Antigen specificity	Effector mechanism	Clinical advantages	Challenge
Tumor-Infiltrating Lymphocytes (TIL)	HLA-dependent	Broad (tumor-associated antigens)	Endogenous TCR-mediated recognition; cytotoxicity via perforin/granzyme and cytokine secretion	Personalized; polyclonal T cell response; effective in antigen-heterogeneous tumors	Limited TIL infiltration; expansion difficulty; local immunosuppression; response variability
Chimeric Antigen Receptor T Cells (CAR-T)	HLA-independent	High (MUC16, mesothelin, FRα)	CAR-mediated surface antigen recognition; cytotoxic T cell activation	Potent cytotoxicity; genetic programmability; MHC-unrestricted	Tumor antigen heterogeneity; poor T cell infiltration; off-tumor toxicity; cytokine release syndrome
TCR-Engineered T Cells (TCR-T)	HLA-dependent	High (NY-ESO-1, MAGE-A4)	Recognize intracellular peptides via engineered TCRs presented by MHC	Target intracellular antigens; broader antigen pool	MHC restriction; tumor MHC-I downregulation; risk of cross-reactivity
Natural Killer (NK) Cells	HLA-independent	Low–medium (non-specific, missing-self recognition)	Natural cytotoxicity; ADCC; cytokine production (IFN-γ, TNF-α)	Innate tumor recognition; low GVHD risk; off-the-shelf potential	Limited persistence; weak tumor homing; suppression by TME cytokines
CAR-NK Cells	HLA-independent	High (engineered CAR specificity)	CAR-directed killing + innate cytotoxicity	Dual killing mechanisms; reduced toxicity vs. CAR-T; allogeneic use	Expansion and persistence; limited clinical data in ovarian cancer
Cytokine-Induced Killer (CIK) Cells	Partially HLA-independent	Low (non-restricted cytotoxicity)	Mixed T/NK-like activity; IFN-γ, granzyme-mediated killing	Easy generation; safe; can be combined with chemo/targeted drugs	Low specificity; limited *in vivo* persistence; modest efficacy
γδ T Cells	HLA-independent	Low–medium (non-peptide phosphoantigen recognition)	MHC-unrestricted cytotoxicity; targeting of stress-induced ligands	Target CSCs; broad recognition; lower risk of immune escape	Difficult *in vitro* expansion; limited clinical experience

## 4 Conclusion

Despite advances in standard therapies, ovarian cancer continues to exhibit poor long-term prognosis due to frequent recurrence, chemoresistance, and an immunosuppressive tumor microenvironment. ACT represents a paradigm shift in cancer immunotherapy by harnessing and enhancing the cytotoxic capacity of immune effector cells, offering targeted and durable responses. Among ACT modalities, TIL therapy has demonstrated early clinical efficacy, CAR-T cell therapy continues to evolve with dual-target and cytokine-enhanced constructs, and NK/CAR-NK cell therapy offers an off-the-shelf alternative with encouraging safety profiles. Furthermore, novel approaches including TCR-T and γδ T cells expand the repertoire of intracellular and non-HLA–restricted targets, while combination strategies with checkpoint inhibitors, metabolic modulators, or conventional therapies show synergistic promise. However, challenges such as tumor antigen heterogeneity, poor cell infiltration, immunosuppressive signaling, and high production costs must still be addressed. Innovations in antigen targeting, gene editing, and cell manufacturing platforms are pivotal for improving efficacy and accessibility. As clinical trials mature and mechanistic insights deepen, ACT is poised to play an increasingly central role in the management of ovarian cancer, moving the field toward more precise, combinatorial, and cost-effective immunotherapeutic solutions.
